# Look out for Your Soft Catheters

**Published:** 1883-03-20

**Authors:** Hiram Nance

**Affiliations:** Kewanee, Ill.


### LOOK OUT FOR YOUR SOFT CATHETERS.

BY HIRAM NANCE, M. D., KEWANEE, ILL.

On the 8th of January, I was telegraphed to from Cambridge, the county seat of Henry county, distance twenty miles, to visit an old acquaintance of mine, R. D. K——, Esq. In the telegram it was stated, "Come, I am afflicted with kidney and bladder disease." I immediately prepared to go, not forgetting to slip in my pocket a medium sized Jaques catheter. I arrived at the house about 4 p. m. and as soon as possible proceeded to examine my patient, and found he had been under the care of one of our little pill fraternity for eight or ten days, or perhaps a longer period. Mr. K—— was aged about 64, the period so common for prostatitis to make its appearance, but it seems that homœopathy had not suspected any mechanical cause for the retention of the urine, which had partially existed since his attack. The patient was suffering terribly, and was taking both homœopathic medicine and morphine in one-eighth grain doses to relieve him. Finding him in a very free state of perspiration, partially stupid, and a strong urinous smell on raising the bed-clothes, and on examining the pubic region it was fully distended, and a continuous stilicidium of urine, I determined at once to introduce a soft catheter, and on doing so, without any pain, I drew off nearly three pints of dark-colored urine. Had this urine been permitted to remain only two or three days longer you all know that he would have died with poison produced from the effects of the absorption of urine, or more properly uremia.

I suspected the cause of the retention before the introduction of the catheter, and called the nurse to the bed-side to show him how to manipulate the instrument, for I presumed on a continuance of the operation for a long time. After prescribing, and indulging in a splendid supper, on the next to the coldest night of the season, I bade good by, feeling happy over the great relief I had been able to give my patient, and feeling confident that the nurse would be able to relieve him with the soft catheter three or four times a day. Imagine my surprise in just twenty-four hours (9 o'clock p. m.) I received a telegram from the Rev. George K——, his son, stating: "Come immediately. Catheter slipped in the bladder." I was horror stricken. I was twenty miles away from patient; Saturday night; no telegraph or trains at that hour, nor would be until Monday; no alternative but to go; mercury near zero; snowing from the West like the deuce, and pouring in our faces. I called in Dr. Nichols, of this place, and solicited his company and advice. We started in the blizzard, and arrived at the house at 1:30 a. m. Sunday. On examination we found the catheter entirely out of sight. They had called in a physician who refused to do anything, saying it would probably require an operation, and as the case was not his he went home.

Visions of lithotomy and lithotripsy flitted hurriedly across my mind; also, the careful introduction of a fine pair of forceps, a hooked wire, stream of water introduced through urethra by syringe, hoping a counter current might expel the intruder. But none of these were tried. We, by feeling the urethra, could detect the end about four or five inches down from the glands penis, and by grasping the urethra and holding it and the catheter and pushing down on the glands we succeeded in moving it forward by jets, as it were, until we could reach it with a pair of nasal forceps. I must say I was rejoiced over our triumph, and many congratulatory remarks were made by the friends over our success.

Never before has such an accident occurred in my practice; nor have I ever read of it occurring to others, and I write this to caution others in the use of this invaluable instrument

MORAL.—When leaving a Jaques catheter with uneducated nurses, always attach a small cord or string to the end, then the instrument can't escape.

